# Digital Support Systems to Improve Child Health and Development in Peru: Protocol for a Randomized Controlled Trial

**DOI:** 10.2196/50371

**Published:** 2023-12-14

**Authors:** Stella Maria Hartinger Pena, Daniel Mäusezahl, Lena Jäggi, Leonel Aguilar, Milagros Alvarado Llatance, Andreana Castellanos, Maria-Luisa Huaylinos Bustamante, Kristen Hinckley, Dana Charles McCoy, Ce Zhang, Günther Fink

**Affiliations:** 1 Swiss Tropical and Public Health Institute Allschwil Switzerland; 2 University of Basel Basel Switzerland; 3 Universidad Peruana Cayetano Heredia Lima Peru; 4 Institute for Computing Platforms Federal Institute of Technology Zurich Zürich Switzerland; 5 Afinidata Fort Collins, CO United States; 6 Harvard Graduate School of Education Cambridge, MA United States

**Keywords:** child development, digital support, parenting, Peru, child, children, childhood, parent, parents, parenting, education, support, parental, cost, low income, low resource, digital health, platforms, pediatric, pediatrics, eHealth, e-health, RCT, RCTs, randomized, controlled trial, development, scalable

## Abstract

**Background:**

Children living in low and middle-income countries (LMICs) are at greater risk for experiencing adversities that can undermine their health and early development. Recently launched digital early childhood development (ECD) programs attempt to support families with young children in their home environments using digital technologies. However, relatively little is known regarding the effectiveness of these new technologies.

**Objective:**

The goal of this study is to rigorously assess the reach, effectiveness, and cost-effectiveness of a newly developed digital ECD platform called Afini. The Afini platform was designed to support parents of young children in low-resource settings to improve ECD and interact with caregivers through messenger services and a chatbot.

**Methods:**

This is a 3-arm cluster randomized controlled trial. In total, 2471 caregivers and their 3- to 9-month-old children were enrolled in the study across 164 study clusters in the San Marcos, Cajabamba, and Cajamarca provinces of Peru. Clusters of participants were randomly assigned to 1 of 3 groups: a control group (72 community clusters and 980 caregiver-child dyads), a home visit intervention group (20 community clusters and 316 caregiver-child dyads), and an Afini intervention group (72 community clusters and 1175 caregiver-child dyads). Families in the control group receive no focused ECD intervention. The home visit group is receiving biweekly home visits by a trained field staff following the national ECD program (*Programa Nacional Cuna Más*) curriculum and training guidelines. Caregivers in the Afini group are receiving ECD activities and advice through the digital platform. The primary study outcome is children’s overall development at the age of 2.5 years, using the internationally validated long form of the Global Scales for Early Development. Secondary outcomes include caregiver engagement; caregiver mental health; screen time; as well as caregiver reports of children’s motor, cognitive, language, and socioemotional development measured through locally piloted and validated tools.

**Results:**

Enrollment started in September 2021 and ended in March 2023. Endline assessments will take place between August 2023 and September 2024.

**Conclusions:**

This study is, to our knowledge, the first to rigorously assess the effectiveness and cost-effectiveness of digital ECD technologies in LMICs. Given the large number of children in LMICs currently receiving only limited external support, the evaluated platform has the potential to improve the short- and long-term well-being of millions of children and their parents globally.

**Trial Registration:**

ClinicalTrials.gov NCT05202106; https://clinicaltrials.gov/ct2/show/NCT05202106

**International Registered Report Identifier (IRRID):**

DERR1-10.2196/50371

## Introduction

According to the World Health Organization, early life adversity affects more than 250 million children younger than 5 years, making it more difficult for them to reach their developmental potential [1,2]. Chronic poverty, malnutrition, and high exposure to infectious diseases undermine children’s development in their early years [1] and limit their physical and cognitive development over time. The most recent estimates suggest that 30% of children younger than 5 years in low and middle-income countries (LMICs) are stunted [3], and 37% of children aged 3-4 years display deficits in their cognitive or socioemotional development [4]. Interventions targeted at this early life stage are crucial for creating environments that support healthy development [5-8] and ensuring children’s long-term economic and overall well-being [9].

Home visiting programs have emerged as one of the most promising and popular approaches for improving child health and well-being in both high- and low-income settings [10], with successful implementation in Bangladesh [11], Brazil [12], Colombia [13], Jamaica [14,15], Pakistan [16], Peru [17], and South Africa [18]. These programs involve training community agents on child development and having them meet with mothers or parents weekly, biweekly, or monthly to discuss their children’s overall well-being. They provide practical guidance to caregivers on creating a healthy and developmentally stimulating environment in the home. Home visiting programs usually have a structured curriculum containing essential child health and development topics that are covered during each home visit. Despite consistently positive impacts on maternal [19,20] and child well-being [21,22], most countries have not adopted home visiting programs at scale due to the high cost and logistical efforts associated with them. In Peru, the state-run national early childhood development (ECD) program (*Programa Nacional Cuna Más* [PNCM]) provides a crèche-based intervention in urban and periurban settings and traditional home visits by trained agents in rural settings [17,23,24].

The rapid rise in cell phone coverage globally [25] and the introduction of mobile computing devices such as smartphones and tablets have created new opportunities for health and social systems to engage with patients and parents at scale [26-28], resulting in a rapid growth in the development of software apps for these platforms [29-34]. Additionally, new mobile phone contracts that offer low-income families access to basic messaging software (such as WhatsApp and Facebook messengers) without formal data contracts have also contributed to the expansion. Such light messenger-only data plans allow users to share information, pictures, and videos on messaging platforms without incurring any cost per message or upload. In our pilot study, 84.4% (152/180) of families owned a smartphone and 94% (169/180) had internet connectivity at home [35]. In this setting, new artificial intelligence (AI)–based ECD technologies using automated chatbots to interact with caregivers of young children seem to be a potentially attractive new option to support parents and their children.

In this project, we will test whether one of these newly developed digital platforms, called Afini [36], can be effective at improving children’s development in a remote population in high-altitude rural Andean Peru. Through this study, we wish to assess the reach, impact, and scalability of the newly developed Afini platform as a tool for improving children’s early development; the subaims are to (1) improve the functionality and usefulness of the Afini platform through qualitative and quantitative feedback and to integrate of state-of-the-art machine learning algorithms, (2) assess the cost and cost-effectiveness of Afini platform as a tool, and (3) disseminate the findings to our targeted population and reach out to the policy makers for further scalability of digital platform initiatives on ECD.

## Methods

### Study Setting

The study is conducted in 3 provinces of the Cajamarca region (San Marcos, Cajabamba, and Cajamarca) in the Northern Andes of Peru. The altitude in the study area ranges between 1900 and 3900 m above sea level. The region is predominantly rural and has a large share of low-resource and remote households engaged in farming, making it representative of many rural and periurban settings in Andean South America. The majority (80%) have regular access to social media platforms [35]. Communities were classified in accordance with the Peruvian National Institute of Statistics and Informatics as urban (communities with >2000 inhabitants with contiguous and grouped homes forming streets) or rural (scattered or grouped houses of up to 2000 inhabitants per community [37]). The study area is partially covered by PNCM.

### Study Design

The study was designed and adapted based on a field-testing phase completed over a period of 5 months from February to July 2021. During the test phase, we conducted a mixed method feasibility pilot to test the reach, reception, and use of the Afini parenting platform. Specifically, we (1) systematically assessed internet connectivity in the greater study region, (2) conducted focus groups and in-depth interviews to adapt Afini to the local cultural context, and (3) assessed use over time and satisfaction with Afini in a sample of 280 mothers with children up to 2 years of age and smartphone access [35].

The study was designed as a 3-arm parallel cluster randomized controlled trial to assess the Afini platform’s effectiveness in improving ECD outcomes relative to a control group receiving no additional intervention as well as relative to a home visit group receiving an ECD home stimulation intervention (gold standard). The trial enrolled 2471 caregivers and their 3- to 9-month-old children across 164 community clusters from September 2021 to March 2023. As described above, communities were randomly assigned to the three study groups: (1) a control group receiving no additional ECD support (72 community clusters and 980 caregiver-child dyads), (2) a home visit intervention group receiving a biweekly home visit by a trained field staff following the national ECD program PNCM curriculum and training guidelines (20 community clusters and 316 caregiver-child dyads), and (3) the Afini intervention group receiving ECD activities and advice through the digital Afini platform (72 community clusters and 1175 caregiver-child dyads).

### Description of the Study Groups and Intervention

#### Control Group

The control group comprises families that receive the regular level of mother and newborn child health care in the region. They have not been benefitting from any ECD program or intervention, but they may have sought assistance regarding parenting from within their network. This study group is only visited once at the beginning (baseline survey) and once at the end of the study (endline survey and assessment).

#### Home Visit Intervention Group

Home visits are considered the gold standard for enhancing child health and development in LMICs [21]. Families in the home visit intervention group (home visit group) receive support through biweekly home visits following the nationally developed and validated curriculum of the national ECD program PNCM. The PNCM program offers home visits to low-income families residing in rural areas (*Servicio de Acompañamiento a Familias*) and daycare services in urban settings (*Servicio de Cuidado Diurno*) [23]. Specifically, all families in the home visit group are receiving biweekly home visits by our trained field staff from when their children turn 6 months until endline assessments at approximately 2.5 years of age. Trained staff members visit families at home every 2 weeks. During these visits, they follow a structured (age-specific) curriculum to educate parents on engaging in educational activities and games for daily interaction with their children, actively play with the child, and provide parents with a set of age-appropriate toys every 3 months. The toys and activities were designed in Peru to promote their children’s psychomotor, cognitive, language, and socioemotional development.

#### Afini Intervention Group

Caregivers in the Afini intervention group (Afini group) were introduced to the Afini digital system on Facebook Messenger during the study baseline visit. During the initial setup, families registered the child’s day of birth and other demographics for Afini to recommend a first educational activity.

After this initial visit, caregivers received automated weekly alerts with personalized content from Afini over Facebook Messenger. Specifically, the Afini system sent weekly reminders to engage in age-specific, health- and development-promoting activities as well as general information on child development and the child's progress. Afini was available for families 24/7, and families could request up to 4 personalized educational activities for their child, ask for advice, or ask any questions related to child development to the Afini chatbot system at any time. The personalization of content works based on the child’s age, initial child development assessment on the platform, and user preferences (ratings) for each educational content.

After approximately 1 year, caregivers in the Afini group will receive a short home visit to ensure they can still connect to the system. Caregivers continue to use Afini independently until they are visited at the end of the study (endline survey and assessment).

The Afini platform is a digital parental support platform for children younger than 5 years in LMIC settings. The platform can be accessed via Facebook Messenger, WhatsApp, or an independent Afini app. Once connected, the Afini content recommender system uses AI to learn about individual children’s needs and recommends content or ideas for activities that caregivers can practice at home together with their child. The platform tracks child development and caregivers can contact the assistant chatbots at any time to receive general advice on child development and parenting. Afini offers over 1500 age-specific, health- and development-promoting activities, articles, and tips to caregivers. Afini’s educational activities, tips, and content are designed in a fun, simple way and with materials that are commonly found at home. All Afini contents offered to caregivers in the Afini group were developed by early development experts and revised by local experts in Cajamarca [35].

### Ancillary Studies

In order to assess whether the usage and impact of Afini can be improved through incentives, a total of 20 study clusters among the 72 Afini intervention clusters were randomly selected for additional top-up intervention. Within these 20 clusters, all caregivers with a prepaid phone plan with either Claro or Bite phone operators were eligible to receive small biweekly data credit transfers with a value of between US $0.8 and US $2. The credit transfers can be used for unlimited access to WhatsApp and Facebook Messenger for 1-3 days, depending on the provider; the monetary value of US $2 corresponds to half a kilo of chicken at a local market. We will assess the marginal impact on the use and development of these incentives as well as their relative cost-effectiveness.

In addition, and to increase the generalizability across the country, we added a comparison group composed of families participating in the existing governmental PNCM program. These families were screened during the enrollment of the main trial and excluded due to their participation in another ECD program; they became eligible for the ancillary study. This PNCM comparison group comprises 237 caregiver-child dyads and is treated the same as a natural control group without intervention. This group was not randomly selected or assigned and will thus mostly be used for descriptive purposes and to provide further context for this study. In summary, the project thus included 2471 trial participants and 237 comparison group participants, totaling 2708 project participants.

### Sample Size Calculations

The study is powered to detect a 0.25 SD difference in normalized child development *z* scores (mean 0, SD 1) at the age 2 years between the Afini group and the control group and a difference of 0.5 SD in *z* scores between the home visit group and the control group. These calculations are based on the following assumptions: (1) an average of 15 (SD 5) children per village or study cluster, (2) an intraclass correlation of 0.12, (3) α=.05, and (4) an attrition rate of 15%. The estimated normalized effect size of 0.5 SD was based on 2 recent reviews of ECD home visiting programs [10,16]. We conservatively anticipate the Afini platform to only reach about half the target population and, thus expect an intention-to-treat effect size of 0.25 SD. Our assumptions regarding intraclass correlations, number of children per cluster, and attrition rates are based on a recently completed ECD-focused study in the same area [17].

### Recruitment

Recruitment occurred continuously over 18 months with a target to enroll 2460 caregiver-child dyads. To identify potential children, the local health facilities’ birth registries from San Marcos, Cajamarca, and Cajabamba were used. All children between 3 and 9 months of age identified during the recruitment period in the study catchment area were eligible. All families that complied with the eligibility criteria were visited in person by study staff and invited to participate. The only reasons for exclusion were if the (1) caregiver and child intended to migrate outside the study area within the next 12 months, (2) child had a diagnosis of congenital abnormalities or a disability such as deafness or blindness (as reported by the caregiver), and (3) child already participated in the national PNCM program and did not belong to the PNCM comparison group. The study was built on a long-standing relationship of the research team with the regional and local health centers that facilitated recruitment.

### Randomization Process

Stratified constrained randomization was used to ensure both spatial and covariate balance across groups. As study strata, 3 study provinces (Cajamarca, Cajabamba, and San Marcos) were used. Within each stratum, communities were allocated with ratios 3.6:1:3.6 for the control group, home visit group, and the Afini group, resulting in a final sample of 72 clusters in both the control group and Afini group and 20 clusters in the home visit group. A total of 1000 potential cluster allocations were randomly drawn using the Stata SE (version 16.0; Stata Corp) software package. We then chose the allocation minimizing mean differences across the 3 arms concerning variables such as cluster population size, cluster number of births, cluster altitude, household cell phone ownership, education of caregiver, and household wealth. Information on these covariates was obtained from governmental sources and validated by our field staff.

### Primary and Secondary Outcome Measures

The primary study outcome will be children’s overall development at the age of 2.5 years. Children’s development will be assessed using the long form of the Global Scales for Early Development (GSED) [38]. The GSED is a new global open access tool designed to provide a standardized method for measuring the development of children up to 36 months of age in low-resource settings. We will use the long form of the GSED tool, which allows for a direct assessment of children’s development through trained assessors.

Secondary outcomes include (1) caregiver-reported early child development in motor, cognitive, language, and socio-emotional domains; (2) quality of a caregiver-child relationship; (3) screen time with and without a child being present; (4) primary female caregiver’s parenting behaviors; (5) parenting attitudes; and (6) mental health. Table 1 summarizes the measures to be collected at baseline and endline—all measures will be applied to the full sample, that is, to all participants independent of their random group assignment. Given that children were very young at baseline (6 months on average), detailed assessments of child development and caregiver-child interactions seemed unlikely to yield much insight at baseline. For overall child development, we used a caregiver report (Caregiver Reported Early Development Instruments [39]) at baseline and will use a more detailed direct observation tool (GSED) at endline. Sociodemographic factors collected at baseline will be used as controls in the final analysis.

**Table 1:** Overview of constructs and measures used at baseline and endline.

Construct and measures		Time of assessment
**Child development (assessment)**		
	Child assessment (GSED^a^ Long Form) [38]	Endline
**Child development (parent report)**		
	CREDI^b^ Long Form [39]	Baseline
	CREDI Short form [39]	Endline
**Parent-child relationship quality**		
	Observation of Mother-Child Interaction [40]—adapted version	Endline
	ARI‐CP 2-5^c^ questionnaire [41] adapted short form	Endline
**Child information**		
	Nutrition, health and health insurance status, participation in ECD^d^ programs	Baseline and endline
**Anthropometrics**		
	Child weight at birth (reported)	Baseline
	Child weight and height (measured)	Endline
**Household demographics**		
	Household composition and assets; education, age, and so on of mother and father	Baseline
**Economic activity**		
	The main occupation for parents, membership in government support programs and COVID-19 support	Baseline
**Negative household events**		
	Household shocks questionnaire	Endline
**Phone network or internet availability and use**		
	Availability of phone, knowledge and use of apps, money spent on the phone, and phone use	Baseline
**Digital literacy**		
	Digital Literacy Scale [42]	Baseline
**Satisfaction with Afini (Afini group) and phone use**		
	Use and satisfaction with Afini and phone use	Endline
**Stimulation materials and experiences**		
	Family Care Indicators [43]	Baseline and endline
**Parenting behaviors, attitudes, and knowledge**		
	Parenting questionnaire (under development)	Endline
**Maternal social support**		
	Social support questionnaire	Baseline and endline
**Maternal mental health**		
	Depression, Anxiety, and Stress scale [44]	Baseline and endline
**Location**		
	GPS data	Baseline and endline

^a^GSED: Global Scales for Early Development.

^b^CREDI: Caregiver Reported Early Development Instruments.

^c^ARI‐CP 2-5: Attachment Relationship Inventory Caregiver Perception 2-5 years.

^d^ECD: early childhood development.

### Data Collection

#### Baseline Assessments

Upon caregivers’ consent, all caregivers completed a baseline survey, including household demographics, household structure, household poverty and income, educational attainment, child assets, household member interactions with the child, caregiver mental health, social support, and digital literacy. The baseline survey also collected data on the child’s age, sex, twin status, gestational length, birthweight, and attendance of daycare or other child- and household-supporting services as well as children’s early development using the Caregiver Reported Early Development Instruments tool [39].

#### End-of-Study Assessments

The endline surveys will occur when children reach 2.5 years of age, which will begin in September 2023. Table 2 shows an overview of the study timeline.

**Table 2:** Study timeline and project activities.

Time period	Description of project activities
July 2020 to January 2021	Study setup, software development, and ethics board approval
February to July 2021	Field testing and finalizing tool development and high-frequency feedback to the programming team
September 2021 to March 2023	Enrollment and baseline survey of 2471 small children and their caregivers in the main trial plus 237 small children and their caregivers in the comparison group (total 2708 project participants)
September 2021 to August 2024	Parenting interventions
August 2023 to September 2024	Endline survey and assessments
October 2024 to February 2025	Data analysis
March 2025	Study dissemination event

The end-of-study assessment will involve gathering information on child development, maternal mental health, mother-child interactions, and parenting attitudes toward the child. To measure the primary outcome, we will use the GSED assessment tool [38]. Secondary outcomes include (1) caregiver-reported early child development in motor, cognitive, language, and socioemotional domains; (2) the quality of a caregiver-child relationship; (3) screen time with and without the child being present; (4) primary female caregiver’s parenting behaviors; (5) parenting attitudes; and (6) mental health. Table 1 provides details on the instruments used.

### Additional Data Collection

To monitor and ensure the quality of the Afini platform, we collect data on users’ platform usage and interactions with the system for the Afini group. These data include activities dispatched to the users, users’ ratings, and feedback on the activities and questions asked. This data access is restricted to the service developers and maintainers and associated with an anonymized internal ID. Through these data, we identify users that have been “recently active” in the platform, which we define as having interacted with the service in any way in the past 15 days. A randomly selected subset of households in the Afini group, qualifying as “recently active,” receives a small biweekly data credit transfer (US $0.8 to US $2.0) to evaluate a potential increase in the use of the platform through small rewards. The data transfer cost and value depend on the service provider (Claro and Bitel) and the aggregator used to perform the credit transfer (Reloadly and DTOne), with these costs increasing within that range from the start of the reward program.

### Home Visits

For each home visit, a short visit form was completed to document the time and date, topics covered, and toys delivered. Additionally, we collected information about the play environment, the toys available, and the interactions between the caregiver and the child at the time of the visit.

Table 3 provides an overview of all study activities. Recruitment and baseline surveys started in the third quarter of 2021. Given the prerandomization of clusters to treatment, interventions were initiated directly after baseline in September 2021, gradually increasing sample sizes in all arms. Recruitment was completed in January 2021, and the first households were scheduled to complete the endline assessment (and exit the study) in September 2023. Interventions will continue (with a steadily decreasing number of households) until the last household completes the endline survey and exits the study in late 2024.

**Table 3:** Overview of study activities^a^.

Study activity		Year and quarter																		
		2021					2022					2023					2024			
		1	2	3	4	1		2	3	4	1		2	3	4	1		2	3	4
**Data collection**																				
	Baseline survey			✓	✓	✓		✓	✓	✓	✓									
	Afini user data collection			✓	✓	✓		✓	✓	✓	✓		✓	✓	✓	✓		✓	✓	
	Home visit logs			✓	✓	✓		✓	✓	✓	✓		✓	✓	✓	✓		✓	✓	
	Endline data collection													✓	✓	✓		✓	✓	
**Interventions**																				
	Home visits			✓	✓	✓		✓	✓	✓	✓		✓	✓	✓	✓		✓	✓	✓
	Afini system use			✓	✓	✓		✓	✓	✓	✓		✓	✓	✓	✓		✓	✓	✓

^a^Summarizes data collection and intervention activities. Each cell corresponds to a quarter in the 2021-2024 period. Active data collection periods and active intervention periods are marked with a ✓.

### Statistical Analysis

After confirming the psychometric reliability and validity of our key variables, we will perform a descriptive analysis (means and percentages) for the main characteristics and calculate prevalence and proportions for the primary outcomes. We will use standard linear regression models to assess the impact of the 2 intervention arms on child development. The primary outcome variable will be overall development as measured by the GSED, normalized to a developmental *z* score with a mean (0, SD 1) in the global reference population. Our primary empirical approach will be intent-to-treat, that is, comparing average outcomes across the 3 arms (independent of intervention compliance) with average outcomes in the control group. We will also conduct additional per-protocol analyses measuring mean differences between compliant children in the intervention arms with compliant children in the control group. For the home visit group, compliance will be defined as receiving at least 18 out of the 36 anticipated visits over the 18-month intervention period. Compliance with the Afini intervention will be defined as having interacted with the system at least once per month for at least 6 months in the observation period. Cluster-robust standard errors will be used to account for the cluster-level randomization of the intervention. Separate models will be estimated with and without baseline covariates.

Specifically, we will assess and control for household factors, caregiver factors, and child factors. Structural equation modeling will be used to test subgroup and mediation effects.

We will calculate the intervention’s cost-effectiveness by comparing the developmental outcomes observed in the 2 intervention arms to the average developmental levels in the control arm to the costs of the 2 interventions per child.

### Ethical Considerations

The study was approved by the University Peruana Cayetano Heredia institutional review board in Lima, Peru (SIDISI: 202522-Ref 030-03-21) and the Ethics Commission for Northwest and Central Switzerland (EKNZ: AO_2021-00002). All participants signed an informed consent before participating in any project-related activity. Verbal consent was asked for key informant interviews and focus group discussions during the pilot phase. The nature of the study was thoroughly explained to all participants, and all questions were answered before obtaining informed consent. Community leaders and local authorities are aware of the study.

## Results

As shown in Table 2, enrollment and baseline data collection were completed in March 2023, and endline assessments were launched in September 2023. Table 4 shows descriptive statistics for the baseline sample by study arm. On average, children were 5 months old at baseline, 49.9% (1223/2452) were female, 8.1% (199/2452) had low birthweight (<2500 g at birth), and 97.5% (2391/2452) of children had a child health card. On average, mothers were 28 years old and considered literate. Fathers were slightly older, with an average age of 31.5 years at baseline, and had slightly higher education levels than mothers. The average household consisted of 2.5 adults and 1 additional child (sibling).

**Table 4:** Descriptive statistics.

Characteristics	Control (n=962)				Home visits (n=315)				Afini (n=1175)		
	Observations, n	Clusters, n	Mean (SE)	Observations, n		Clusters, n	Mean (SE)	Observations, n		Clusters, n	Mean (SE)
Child age (months)	959	67	5.0 (0.11)	315		19	5.1 (0.22)	1169		78	5.1 (0.10)
Child is female	954	67	0.51 (0.02)	314		19	0.48 (0.03)	1172		78	0.50 (0.01)
Child has a health card	959	67	0.98 (0.01)	315		19	0.97 (0.01)	1169		78	0.98 (0.01)
Child born <2500 g	949	67	0.08 (0.01)	308		19	0.10 (0.02)	1152		78	0.08 (0.01)
CREDI^a^ score	950	67	43.0 (0.066)	307		19	43.0 (0.163)	1163		78	43.2 (0.065)
Mother’s age (years)	945	67	27.7 (0.26)	311		19	28.6 (0.48)	1155		78	28.0 (0.22)
Mother is literate	941	67	0.98 (0.01)	307		19	0.97 (0.01)	1151		77	0.99 (0.004)
Mother education level	944	67	3.2 (0.08)	310		19	3.2 (0.23)	1154		78	3.4 (0.08)
Father’s age (years)	843	66	31.2 (0.26)	277		18	32.1 (0.52)	1027		76	31.6 (0.26)
Father is head of household	844	66	0.94 (0.01)	277		18	0.94 (0.02)	1026		76	0.94 (0.01)
Father education level	832	66	3.5 (0.07)	267		18	3.5 (0.22)	1007		76	3.7 (0.07)
SES^b^ index	957	67	2.9 (0.12)	315		19	3.1 (0.29)	1169		78	3.2 (0.12)
Stimulation index	868	66	3.4 (0.06)	285		19	3.4 (0.14)	1076		78	3.5 (0.06)

^a^CREDI: Caregiver Reported Early Development Instruments.

^b^SES: socioeconomic status.

We anticipate completing the endline assessments in September 2024 and presenting final trial results to the local and national governments in Peru in early 2025.

## Discussion

### Principal Findings

In this study, we present the design and setup of a cluster randomized trial to evaluate the effectiveness and cost-effectiveness of Afini, a digital platform that provides digital support to children from 0 to 7 years of age. The trial compares the developmental improvement achievable through the Afini platform to developmental improvements achievable through home visits, which currently are considered the gold standard in this field. While home visits offer the advantage of personal interactions with caregivers, the main benefits of new technologies, such as Afini, lie in their flexibility. Using a chatbot and AI system, Afini efficiently and automatically takes into account the caregiver’s and child’s preferences. It then offers age-specific recommendations for development-promoting activities that parents can undertake with their children whenever they have the time for such interactions. The platform also provides advice on early child development, well-being, and health similar to the contents typically delivered through ECD home visits.

The proposed trial is innovative and timely for several reasons. Due to the rapid global increase in cell phone coverage [25], there are now unprecedented opportunities for health and education systems to interact with patients and families and intervene at scale effectively and efficiently [31,33,34]. These advances have paved the way for the development and use of parenting support platforms as a means of engagement [26,30,43,45,46]. However, several key questions must be addressed before such platforms can be used in remote and resource-constrained settings. These include understanding their reach and actual impact on child development. Additionally, it remains unclear whether these technologies can be scaled to populations with limited digital literacy.

Understanding the extent to which digital platforms can reach low-income communities and families is crucial for understanding the feasibility and effectiveness of digital platforms in addressing the needs of vulnerable populations. During our formative research, we were particularly concerned about the challenges related to technology access, internet connectivity, and affordability. However, our early findings indicated that the large majority of mothers with young children had internet and smartphone access. Furthermore, 2 months after baseline, 84% of mothers reported using the platform at least once, and 87% rated it as useful [35]. Similar studies conducted in LMICs have also reported high acceptance of digital parenting interventions [47,48] and good reach for mobile health interventions to improve maternal and child health [31,34]. However, the majority of existing data on digital parenting support interventions during early childhood has been collected in high-income countries [27,30,33,43]. While those studies show promise in parenting outcomes and engagement, such as enhancing parental self-efficacy [49,50], data from LMIC contexts are largely missing. Furthermore, to date, limited data exist on digital parenting interventions’ impact on child development, with few randomized controlled trials [51], and many studies still in the piloting stages, with small samples, or in ongoing data collection [52-54].

Our second question revolves around the impact of digital platforms on caregiver-child interactions and child development in low-resource communities. Caregiver-child interactions are vital for a child’s early development, and it is important for a digital platform targeting households in low-resource communities to facilitate positive and developmentally appropriate caregiver-child interactions. A study conducted in the United States examined the availability and quality of the apps in app stores and found very few apps offering tailored materials to parents instead of promoting generic guidance that may have limited usefulness in certain contexts [55]. However, several pilot studies demonstrated that digital interventions are feasible, engaging, and cost-effective ways for delivering developmental education to families, particularly in enhancing language-promoting behaviors [56,57]. A recent review highlighted the potential of mobile health and apps to promote early language development as well, although it raised concerns about the frequency and consistency with which parents will access and use digital interventions in their daily lives [58].

From a policy perspective, scalability is maybe the most critical aspect to consider when evaluating a digital platform. It is important to assess whether the platform can be successfully extended to other settings and populations beyond the initial study in the rural Andes. This becomes especially relevant when there is a possibility of limiting access for subsets of the population with varying levels of digital literacy, particularly in the absence of guidelines or follow-up assistance. Initial data collected in the study area showed that smartphone access and connectivity are almost universal [35]. While there have been limited studies demonstrating scalability thus far, it is essential to identify any unanticipated limitations or challenges that may arise during implementation and adherence, particularly in populations with diverse levels of digital literacy.

The proposed study has some limitations. First, while all intervention content was carefully adapted and is thus optimized for the local context studied, the materials used (and results obtained) may not directly work and translate to other settings. Second, our study is only able to follow-up children for about 2.5 years and can thus only give short-term treatment effects that may not fully capture long-term gains for treated children. Third, our intervention focused primarily on a broad measure of child development and did not offer specific insights into more narrow areas of early childhood health such as nutrition or emotional development. Future studies will be needed to address these issues.

### Conclusions

This trial will provide valuable insights into the impact and scalability of tailored digital ECD platforms, particularly in reaching remote and vulnerable populations in low- to middle-income settings, and address challenges faced by home visiting models, such as cost and sustainability. We wish to demonstrate the platform’s effectiveness, especially among diverse population groups with varying levels of digital literacy. This is crucial for designing effective future interventions.

## Abbreviations

AI: artificial intelligence
ARI-CP 2-5: Attachment Relationship Inventory Caregiver Perception 2-5 years
ECD: early child development
GSED: Global Scales for Early Development
LMIC: low and middle-income countries
PNCM: Programa Nacional Cuna Más

## Data Availability

The data sets generated and analyzed during this study are available from the corresponding author on reasonable request. All trial data will be made publicly available upon completion of the trial and analysis.
